# Severe diaphragmatic dysfunction with preserved activity of accessory respiratory muscles in a critically ill child: a case report of failure of neurally adjusted ventilatory assist (NAVA) and successful support with pressure support ventilation (PSV)

**DOI:** 10.1186/s12887-019-1527-2

**Published:** 2019-05-17

**Authors:** Thomas Langer, Serena Baio, Giovanna Chidini, Tiziana Marchesi, Giacomo Grasselli, Antonio Pesenti, Edoardo Calderini

**Affiliations:** 1 Department of Pathophysiology and Transplantation, University of Milan, Milan, Italy; 2 Fondazione IRCCS Ca’ Granda Ospedale Maggiore Policlinico, Anestesia e Terapia Intensiva Donna-Bambino, Milan, Italy; 3 Department of Anesthesia, Critical Care and Emergency, Fondazione IRCCS Ca’ Granda Ospedale Maggiore Policlinico, Milan, Italy

**Keywords:** Ventilators, mechanical, Interactive ventilatory support, Diaphragm, Respiratory system, Dyspnea, Control of breathing, Case report

## Abstract

**Background:**

Neurally adjusted ventilatory assist (NAVA) is an alternative to pressure support ventilation (PSV) potentially improving patient-ventilator interaction. During NAVA, diaphragmatic electrical activity (EAdi) is used to trigger the ventilator and perform a proportional respiratory assistance. We present a case in which the presence of severe bilateral diaphragmatic dysfunction led to a failure of NAVA. On the contrary, the preserved activity of the accessory inspiratory muscles allowed a successful respiratory assistance using PSV.

**Case presentation:**

A 10-year-old girl developed quadriplegia after neurological surgery. Initially, no spontaneous breathing activity was present and volume controlled ventilation was necessary. Two months later spontaneous inspiratory efforts were observed and a maximal negative inspiratory force of − 20 cmH_2_O was recorded. In addition, a NAVA nasogastric tube was placed. The recorded EAdi signal, despite showing a phasic activity, had a very low amplitude (1–2 μV). Two brief (15 min) breathing trials to compare PSV (pressure support = 8 cmH_2_O) with NAVA (Gain = 5 cmH_2_O/μV, inspiratory trigger = 0.3 μV) were performed. On PSV, the patient was well adapted with stable tidal volumes, respiratory rates, minute ventilation, end-tidal and venous carbon dioxide levels. When switched to NAVA, her breathing pattern became irregular and she showed clear sign of increased work of breathing and distress: tidal volume dropped and respiratory rate rose, leading to an increase in total minute ventilation. Nevertheless, end-tidal and venous carbon dioxide rapidly increased (from 49 to 55 mmHg and from 52 to 57 mmHg, respectively). An electromyographic study documented an impairment of the diaphragm with preserved activity of the accessory inspiratory muscles.

**Conclusions:**

We document the failure of mechanical assistance performed with NAVA due to bilateral diaphragmatic dysfunction in a critically ill child. The preserved activity of some accessory respiratory muscles allowed to support the patient effectively with pressure support ventilation, i.e. by applying a pneumatic trigger. The present case underlines (i) the importance of the integrity of the respiratory centers, phrenic nerves and diaphragm in order to perform NAVA and (ii) the possible diagnostic role of EAdi monitoring in complex cases of weaning failure.

## Background

In the intensive care unit (ICU), mechanical ventilatory assistance and weaning attempts are frequently performed with pressure support ventilation (PSV). During PSV, inspiration is triggered by flow or pressure changes in the respiratory circuit, induced by the patient’s inspiratory efforts.

Neurally adjusted ventilatory assist (NAVA) has been recently proposed as an alternative to PSV [1]. The use of NAVA requires the continuous recording of the electrical activity of the crural diaphragm (EAdi, expressed in microVolts - μV) through a dedicated esophageal probe. The EAdi is the temporo-spatial summation of the action potentials, from the respiratory centers to the diaphragm motor units. It correlates with the global inspiratory effort both in healthy subjects and in patients with respiratory failure, and is therefore considered to provide a reliable estimate of the central respiratory drive [2]. During NAVA, the magnitude of inspiratory pressure assistance is continuously regulated through the EAdi, according to a proportionality factor called NAVA gain (expressed in cmH_2_O/μV). In addition, the EAdi signal acts also as inspiratory/expiratory trigger. Several studies have shown that NAVA, as compared to PSV, significantly improves patient-ventilator synchrony [3–5], therefore favoring a successful weaning from mechanical ventilation.

Clearly, proper functioning of NAVA is strictly linked to the integrity of the respiratory centers, phrenic nerves and diaphragmatic muscle fibers. Indeed, in case of preserved respiratory drive but altered phrenic nerve conduction, the resulting low EAdi signal is not representative of the inspiratory efforts, and is therefore not suitable to trigger respiratory assistance. For example, a partial neuromuscular blockade, while likely causing an increase in central respiratory drive, is associated with a significant reduction in EAdi [6].

We describe here a patient with severe bilateral diaphragm dysfunction leading to a clear failure of ventilatory assistance performed with NAVA. On the contrary, respiratory assistance performed with PSV was successful, due to the preserved activity of the accessory inspiratory muscles.

This case report was prepared following the CARE Guidelines [7].

## Case presentation

A 10-year-old Caucasian girl, weighing 45 kg, underwent an endoscopic transnasal craniotomy to remove an adamantinomatous craniopharyngioma. After the apparently uneventful operation, the patient developed postoperative encephalitis, obstructive hydrocephalus, intracranial hypertension and became comatose. A ventriculoperitoneal shunt was placed to limit intracranial hypertension. The patient awoke from the coma with flaccid quadriplegia, likely due to tonsillar herniation. Since she had no spontaneous breathing activity, a tracheostomy was performed, and she was ventilated with volume controlled ventilation.

Two months thereafter, the patient was transferred to our pediatric ICU to continue the treatment and evaluate the possibility of pursuing a respiratory weaning.

Compliance of the respiratory system (0.8 ml/cmH_2_O/kg), PaO_2_-to-FiO_2_ ratio (490 mmHg) and a negligible alveolar dead space fraction were measured. Once mechanical support was reduced, spontaneous inspiratory efforts were observed. A maximal negative inspiratory force (NIF) of − 20 cmH_2_O (Servo-I ventilator, Maquet, Solna, Sweden) was recorded. In addition, a NAVA nasogastric tube (Maquet Critical Care AB, Solna, Sweden) was positioned to measure EAdi signal and to start NAVA ventilation, with the aim of improving patient-ventilator synchrony. The correct placement of the NAVA probe was carefully checked using the positioning window of the ventilator and the ECG signals [8].

At a preliminary evaluation, the patient appeared poorly adapted to NAVA, even in the presence of a very high NAVA gain (5 cmH_2_O/μV) and a sensitive neural trigger (0.3 μV). The recorded EAdi signal, despite showing a phasic activity, had a very low amplitude.

On the contrary, she appeared well synchronized and adequately supported on PSV. We therefore decided to perform two brief (15 min) “breathing trials” to compare PSV with NAVA, in order to understand the possible clinical implications of these observations. The following ventilatory settings were applied:

1. Pressure support ventilation with pressure support of 8 cmH_2_O, flow trigger sensitivity set at 4 and expiratory trigger set at 25% of peak inspiratory flow.
2. Neurally-adjusted ventilatory assistance with NAVA gain of 5 cmH_2_O/μV and neural inspiratory trigger of 0.3 μV. Of note, during NAVA, inspiratory assist can also be triggered by a pneumatic signal in case of failure of the neural trigger.

A constant positive end-expiratory pressure of 4 cmH_2_O and fraction of inspired oxygen of 0.21 were applied throughout the clinical test. We recorded ventilatory variables (tidal volume, respiratory rate, mean airway pressure, end tidal carbon dioxide, pulse oximetry), EAdi and heart rate at 3 min intervals during both phases. A blood gas analysis of central venous blood and non-invasive blood pressure were measured at the beginning and at the end of each 15-min trial.

Table 1 summarizes the observed results. On PSV, the patient presented stable tidal volumes, respiratory rates, minute ventilation, end-tidal and venous CO_2_ levels. On the contrary, when switched to NAVA, an immediate drop in tidal volume with consequent marked reduction in minute ventilation was observed. Thereafter, the patient increased the respiratory rate from 15 to 24 breaths per minute, leading to an increase in total minute ventilation, despite the reduced tidal volume. Nevertheless, end-tidal CO_2_ progressively increased from 49 to 55 mmHg with a corresponding increase in venous CO_2_ from 52 to 57 mmHg. Peripheral arterial oxygen saturation, as measured with pulse oximetry, did not change. The amplitude of the EAdi signal remained very low during both phases and it did not change significantly with the ventilation mode. Of note, during NAVA, the majority of breaths were initiated by the secondary source, i.e. by the pneumatic trigger, as the EAdi signal was frequently not able to trigger inspiration. Clinically, while the patient was well adapted to PSV, on NAVA her breathing pattern became irregular. She showed clear signs of increased work of breathing and distress, such as nasal flaring and suprasternal-intercostal retractions, mild tachycardia and hypertension.

**Table 1:** Pressure support ventilation and NAVA

	Ventilation	0’	3’	6’	9’	12’	15’
Vt [ml]	PS	150	180	165	170	170	180
	NAVA	80	88	110	95	100	86
RR [bpm]	PS	16	18	17	16	16	17
	NAVA	15	22	20	22	24	24
MV [l/min]	PS	2.7	2.7	2.7	2.8	2.8	2.5
	NAVA	1.2	2.4	3	3.2	2.5	2.8
MAP [cmH_2_O]	PS	12	12	12	12	12	12
	NAVA	8	7	10	10	7	11
EtCO_2_ [mmHg]	PS	47	47	48	47	47	48
	NAVA	49	53	52	53	55	55
EAdi max [μV]	PS	1.6	1.7	2.1	1.1	1.3	1.5
	NAVA	1.7	1.8	2	1.7	1.2	1.5
EAdi min [μV]	PS	0.9	0.4	0.4	0.3	0.3	0.4
	NAVA	0.6	0.2	0.5	0.4	0.5	0.2
HR [bpm]	PS	130	129	123	123	121	117
	NAVA	114	123	125	126	123	122
SBP/DBP [mmHg]	PS	140/90					140/96
	NAVA	110/99					150/110

*Definition of abbreviations: PSV* pressure support ventilation, *NAVA* neurally adjusted ventilatory assistance, *V*_*T*_ tidal volume, *RR* respiratory rate, *MV* minute ventilation, *MAP* mean airway pressure, *E*_*T*_*CO*_*2*_ end tidal carbon dioxide, *EAdi* electrical activity of the diaphragm, *HR* heart rate, *SBP/DBP* systolic blood pressure/diastolic blood pressure

An electromyographic study was performed and the presence of a sensory-motor polyneuropathy compatible with critical illness polyneuropathy was documented. The muscles of the head and neck showed less involvement. An involvement of the diaphragm and of the trapezius was documented, while other accessory muscles, such as the sternocleidomastoid and the intercostal muscles showed a preserved activity.

A magnetic resonance imaging of the brain and spinal cord revealed the presence of a lesion of the posterolateral region of the high spinal cord, compatible with the previous infective process and/or hypertensive damage.

One month thereafter, the patient was transferred to a long-term rehabilitation center. While the upper limbs recovered some muscular strength, she was still completely ventilator-dependent 6 months after discharge from our pediatric ICU. EAdi measurements were not repeated.

## Discussion and conclusions

The breathing rhythm originates from the respiratory centers, located in the medulla. The ideal mechanical assistance of spontaneous ventilation should thus employ, as respiratory trigger, a signal directly originating from these centers. While this is currently not feasible, EAdi is considered a valid peripheral surrogate. Indeed, the EAdi signal results from (i) the activation of the respiratory centers, (ii) the conduction of the electric signal through the nuclei of the phrenic nerve, the phrenic nerves and the neuromuscular junction and, finally, (iii) the activation of the muscular fibers of the diaphragm. Therefore, any pathological process involving the generation and conduction of the impulse from the respiratory centers to the muscular fibers of the diaphragm could interfere with the generation of the EAdi signal.

On the one hand, a direct impairment of the respiratory centers could stop their activity. In this case, the resulting lack of an EAdi signal would correctly identify the absent respiratory drive [9]. On the other hand, in case of a more peripheral impairment, such as a lesion to the phrenic nuclei, phrenic nerve and/or neuromuscular junction, the reduced/absent EAdi signal does not indicate a reduced/absent respiratory drive.

Our patient experienced several pathological conditions potentially affecting her breathing activity. First, the intracranial hypertension leading to tonsillar herniation could have compromised directly the respiratory centers in the medulla. Second, the tonsillar herniation and the infectious process itself could have caused a lesion of the cervical spinal cord (documented with MRI) potentially involving the nuclei of the phrenic nerve. Third, an impairment of the phrenic nerves due to critical illness polyneuropathy is certainly conceivable [10]. Finally, the muscular fibers of her diaphragm could be atrophic due to the prolonged mechanical assistance [11].

In our case, the measurement of EAdi was a useful diagnostic tool to identify a severe bilateral diaphragmatic dysfunction. Indeed, while EAdi showed a phasic activity, its amplitude was very small, potentially suggesting a neuromechanical uncoupling between the respiratory centers and the diaphragm. Several studies have investigated and quantified the interaction between the respiratory motor neurons arising from the respiratory centers and the respiratory muscles, i.e. neuromechanical coupling.

Some studies focused on the major respiratory muscle, the diaphragm, measuring the variation in transdiaphragmatic pressure (abdominal – esophageal pressure) and dividing the pressure swing by the variation in EAdi [12]. In this way, an index was generated which expressed the transdiaphragmatic pressure generated by each microvolt of EAdi. This index was called diaphragm neuromechanical coupling [12].

Other studies, instead, focused on the pressure generated by all respiratory muscles, using either the variation in airway [13] or esophageal [14] pressure generated during an inspiratory effort against an occluded airway. This pressure swing divided by the variation in EAdi was termed either Neuromechanical efficiency or Pmusc/Eadi index (PEI), according to the used proxy of muscular function (airway pressure vs. esophageal pressure).

A typical condition of low neuromechanical coupling, regardless of the applied definition, is spinal muscular atrophy. Indeed, high EAdi variations and tonic diaphragmatic activity throughout the respiratory cycle (EAdi 25–50 μV) with very low muscular effort have been described in this context [9]. Our patient, on the contrary, had diaphragmatic dysfunction *and* low EAdi. While not directly measured, we can therefore speculate that our patient could have had a slightly lower or even a normal diaphragm neuromechanical coupling. Interestingly, given the preserved activity of the accessory respiratory muscles, the Neuromechanical efficiency or Pmusc/Eadi index could have been paradoxically high. Indeed, due to the preserved activity of the accessory respiratory muscles, the patient was able to achieve variations in airway pressure as high as 20 cmH_2_O, despite minimal EAdi swings (1–2 μV). Of note, the EAdi levels observed in our case are slightly lower than normal values reported in the literature [15].

Since NAVA delivers mechanical assistance proportionally to the EAdi signal, the low registered values caused a reduction of tidal volume, even in the presence of a very high NAVA gain (5 cmH_2_O/μV). Despite the increase in respiratory rate, and therefore of minute ventilation, carbon dioxide continued to rise during NAVA, likely due to an increased fraction of anatomical and instrumental dead space ventilation. Finally, it is worth underlining that the majority of inspiratory acts during NAVA were not triggered by the EAdi signal, but by the secondary pneumatic trigger, as neural signals were frequently insufficient to initiate the breath. Of note, during NAVA, the level of inspiratory assist of pneumatically-triggered breaths remains proportional to the recorded EAdi signal. This might therefore result in an insufficient mechanical assistance, and consequently low tidal volumes, in cases of low EAdi amplitude.

As compared to NAVA, our patient was adequately supported with PSV: the pneumatic trigger of PSV was effectively activated by the preserved, or possibly augmented [16] activity of the accessory inspiratory muscles.

A similar clinical case, describing an 8-year-girl with tetraplegia due to a hemorrhagic transformation of a large C3-C4 medullary arteriovenous malformation has been recently described [17]. The authors report, as we did, an initial presence of small EAdi variations. However, differently from our case, the EAdi signal was able to trigger the ventilator and thus NAVA could be performed successfully. Of note, the activity of the accessory respiratory muscles was not assessed in this case.

Finally, it is worth mentioning that we did not perform an ultrasound evaluation of the diaphragm, which is certainly an emerging non-invasive bedside tool for the assessment of its function [18, 19].

In conclusion, we documented the failure of mechanical assistance performed with NAVA due to partial, bilateral diaphragmatic dysfunction. At the same time, the preserved activity, and potentially augmented strength, of some accessory respiratory muscles, allowed to support the patient effectively with pressure support ventilation, i.e. by applying a pneumatic trigger and a fixed preset level of inspiratory assist.

The present case underlines the potential use of EAdi measurements for the differential diagnosis of diaphragmatic dysfunction. Furthermore, it highlights the limits of EAdi to trigger the ventilator and to perform a proportional ventilatory assistance, in case of diaphragmatic dysfunction.

## Abbreviations

cmH_2_O: Centimetres of water
CO_2_: Carbon dioxide
EAdi: Electrical activity of the diaphragm
mmHg: Millimetres of mercury
NAVA: Neurally adjusted ventilatory assist
PaO_2_: Arterial partial pressure of oxygen
PaO_2_-to-FiO_2_ ratio: Ratio between arterial partial pressure of oxygen and fraction of inspired oxygen
PSV: Pressure support ventilation
μV: Microvolts
